# Commentary on: Effects of Lumbar Discectomy on Disability and Depression in Patients With Chronic Low-Back Pain

**DOI:** 10.5812/aapm.2227

**Published:** 2012-07-10

**Authors:** Gun Choi

**Affiliations:** 1 Department of Medicine, Hanyang University of Medical Sciences, Seoul, South Korea

**Keywords:** Depression, Discectomy, Low Back Pain

Dear Editor,

In your June 2011 issue, we found an interesting study by Farzanegan et al. entitled, “Effects of lumbar discectomy on disability and depression in patients with chronic low-back pain.” (1). The authors demonstrated the beneficial role of surgery in chronic back pain for patients with disability and depression. Patients with chronic back pain are usually also depressed (2). Evaluating these patients is difficult. Evidence is currently lacking to recommend optimal methods to evaluate these patients. Farzanegan et al. used the Beck depression inventory to evaluate depression and Rolland and Morris’s questionnaire to measure disability. These are simple, fast, and reliable methods for screening back pain in the general population (3, 4). Depression and chronic pain have been thought to worsen the prognosis of low-back surgery. Thus, patients with depression spend more time in diagnosis and receive more surgical interventions. Researchers have recommended screening for emotional distress and aggressively treating psychological issues before surgery (3). Contrary to what intuition might tell us, these precautions could actually increase the chronicity of pain and lead to poorer results after surgery.

The association between depression and pain after low-back surgery is not consistent across studies. Depression and disability have been found to be highly influential in patients undergoing lumbar spine surgery. The psychological profile of depression has predicted poorer outcomes in patients with chronic sciatica pain and disability (5). The study by the Swedish lumbar spine society was not able to demonstrate an association between depressive symptoms and surgical outcomes and concluded that depressive symptoms should not be a contraindication to surgery (6). The results of lumbar surgery are influenced by depression and duration of pain. A systemic review found that depression usually begins after the onset of pain (7). Patients with chronic pain should be screened for depression, especially because these patients do not show the classical signs of depression. For depressed patients, the treatment of low-back pain should be multifaceted, and lumbar surgery should be an integral part of the treatment plan. This fact is validated by the present study. Postsurgery results are better when a structural cause is found, such as in the authors’ sample, which included only patients with herniated discs. Low-back surgery should not be delayed when a structural cause is present in depressed patients because both depression and duration would influence the outcomes of surgery.
